# Basal localization of MT1-MMP is essential for epithelial cell morphogenesis in 3D collagen matrix

**DOI:** 10.1242/jcs.135236

**Published:** 2014-03-15

**Authors:** Sarah A. Weaver, Brit Wolters, Noriko Ito, Anna M. Woskowicz, Kazuyo Kaneko, Yasuyuki Shitomi, Motoharu Seiki, Yoshifumi Itoh

**Affiliations:** 1 Kennedy Institute of Rheumatology, University of Oxford, Roosevelt Drive, Oxford OX3 7FY, UK; 2 Division of Cancer Cell Research, Institute of Medical Science, The University of Tokyo, 4-6-1 Shirokanedai, Minato-ku, Tokyo, 108-8639, Japan

**Keywords:** MT1-MMP, TGFβ, Collagen, Epithelial cells, Tubulogenesis

## Abstract

The membrane-anchored collagenase membrane type 1 matrix metalloprotease (MT1-MMP) has been shown to play an essential role during epithelial tubulogenesis in 3D collagen matrices; however, its regulation during tubulogenesis is not understood. Here, we report that degradation of collagen in polarized epithelial cells is post-translationally regulated by changing the localization of MT1-MMP from the apical to the basal surface. MT1-MMP predominantly localizes at the apical surface in inert polarized epithelial cells, whereas treatment with HGF induced basal localization of MT1-MMP followed by collagen degradation. The basal localization of MT1-MMP requires the ectodomains of the enzyme because deletion of the MT-loop region or the hemopexin domain inhibited basal localization of the enzyme. TGFβ is a well-known inhibitor of tubulogenesis and our data indicate that its mechanism of inhibition is, at least in part, due to inhibition of MT1-MMP localization to the basal surface. Interestingly, however, the effect of TGFβ was found to be bi-phasic: at high doses it effectively inhibited basal localization of MT1-MMP, whereas at lower doses tubulogenesis and basal localization of MT1-MMP was promoted. Taken together, these data indicate that basal localization of MT1-MMP is a key factor promoting the degradation of extracellular matrix by polarized epithelial cells, and that this is an essential part of epithelial morphogenesis in 3D collagen.

## INTRODUCTION

Epithelial cells are essential components of fundamental structures of multicellular organisms. They form a sheet attached to their own extracellular matrix (ECM), the basal lamina, which divides tissues into different compartments. Epithelial cells adhere tightly to one another to form these epithelial sheets. The tight junction is a specialized intercellular junctional complex that mediates adhesion between epithelial cells (Pieczynski and Margolis, 2011; St Johnston and Sanson, 2011). It serves to form tight seals between adjacent epithelial cells, and also creates the boundary between the apical and basolateral membrane domains of a cell, acting as a barrier to intramembranous diffusion of proteins and macromolecules. Thus, the tight junction provides cells with different functionalities between the apical and basolateral surfaces (Pieczynski and Margolis, 2011; St Johnston and Sanson, 2011). According to this polarization, vesicle transport of secretory proteins and membrane proteins are regulated in a precise manner. For instance, peptide hormones and growth factors are secreted to the apical surface, whereas extracellular matrix components – such as laminins and type IV collagens – are secreted to the basal side (Pieczynski and Margolis, 2011; St Johnston and Sanson, 2011).

One important function of epithelial cells is to create tubules that provide functionality to different organs. However, the mechanism of tubulogenesis is not clearly understood. As a prototypical model-system of epithelial tubulogenesis, Madin-Darby canine kidney (MDCK) epithelial cells have been widely utilized (Kadono et al., 1998; Montesano et al., 1991a; Montesano et al., 1991b; Pollack et al., 1998). In this model, cells are cultured in a 3D collagen gel where they form branching tubules upon stimulation with hepatocyte growth factor (HGF) (Kadono et al., 1998; Montesano et al., 1991a; Montesano et al., 1991b; Pollack et al., 1998). The cells establish epithelial polarity and form a lumen inside the structure; to extend tubules into a 3D collagen gel, cells degrade the surrounding collagen matrix. One of the membrane-anchored matrix metalloproteinases (MMPs) MT1-MMP (also known as MMP14), plays an essential role in this process (Kadono et al., 1998). When the MT1-MMP gene is downregulated by antisense oligonucleotides, MDCK cells no longer form tubules in collagen gels. Overexpression of MT1-MMP, however, has been shown to deform tubule structures, leading to the formation of enlarged cysts due to excess degradation of the collagen matrix (Hotary et al., 2000; Kadono et al., 1998). These findings clearly link degradation of pericellular ECM by MT1-MMP with epithelial morphogenesis. However, it is not understood how MT1-MMP is regulated during tubulogenesis.

MT1-MMP was the first transmembrane-type MMP to be discovered. It degrades various components of the extracellular matrix on the cell surface, including collagen-I, -II, -III, fibronectin, vitronectin, laminins and aggrecan core protein (Ohuchi et al., 1997), and also activates pro-MMP2 (Sato et al., 1994) and pro-MMP13 (Knäuper et al., 1996), expanding its proteolytic repertoire on the cell surface. Furthermore, MT1-MMP processes cell-surface molecules, including CD44 (Kajita et al., 2001), syndecan1 (Endo et al., 2003), αV integrin (Ratnikov et al., 2002) and low-density lipoprotein receptor-related protein 1 (LRP1) (Rozanov et al., 2004), affecting cellular functions, especially cell motility. Among these enzymatic activities, collagenolytic activity has been proposed to be particularly important. MT1-MMP-null mice exhibit several developmental phenotypes, including skeletal abnormality and soft tissue fibrosis, and these phenotypes are, at least in part, due to a lack of pericellular collagen degradation (Holmbeck et al., 1999; Zhou et al., 2000). MT1-MMP is also an essential pericellular collagenase for cancer cells to grow in a collagen-rich environment (Hotary et al., 2003). MT1-MMP also plays a major role in angiogenesis (Chun et al., 2004; Hiraoka et al., 1998), in migration of smooth muscle cells to the neo-intima of the artery upon injury (Filippov et al., 2005) and in cartilage invasion by rheumatoid synovial tissue (Miller et al., 2009; Sabeh et al., 2010). Thus, MT1-MMP plays important roles in invasive processes of different cell types.

When a single cell migrates in a 3D matrix, MT1-MMP is localized at the leading edge of the cell to generate a path for cell migration. However, in multicellular structures, such as tubules in a 3D matrix, localization becomes more complex as it is essential for a population of cells to have coordinated localization of MT1-MMP in order to extend into the 3D matrix. Therefore, we postulated that the spatiotemporal regulation of MT1-MMP activity across the different components of a tubule is an important part of the morphogenic program of epithelial cells. In inert, well-polarized epithelial cells the default pathway of MT1-MMP secretion was found to be towards the apical surface. Stimulation of the cells with HGF induced localization of MT1-MMP to the basal surface, leading to a substantial increase in collagenolytic activity at the basal side. We also found that transforming growth factor β (TGFβ) plays a role in efficient basal localization of MT1-MMP, as well as tubulogenesis. Our data indicate that localization of MT1-MMP at the basal side is an essential part of epithelial tubulogenesis in 3D collagen matrices.

## RESULTS

### MT1-MMP distribution in the polarized epithelial cell layer

**Fig. 1.**
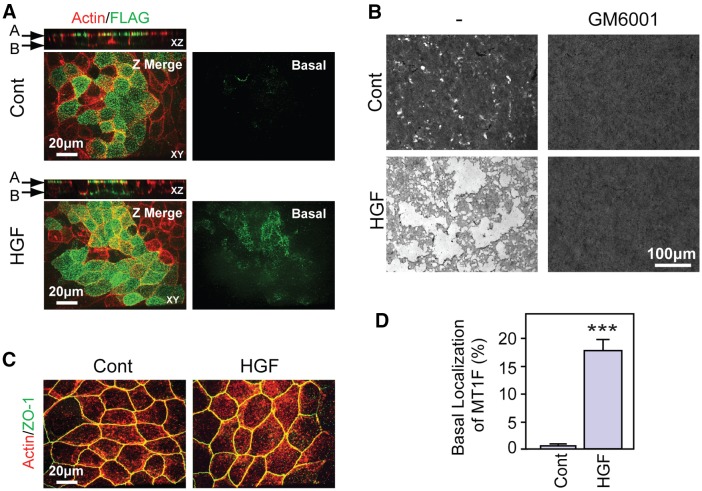
**Change in MT1-MMP localization by HGF.** (A) MDCK cells expressing FLAG-tagged MT1-MMP (MT1F) were cultured on collagen-coated Transwell inserts and treated with or without HGF (50 ng/ml) for 24 h. Cell surface localization of MT1-MMP was detected with the FLAG M2 antibody (green). Cells were also counter-stained for actin (red). Merged XY section (XY), single XZ section (XZ) or single basal surface section (Basal), obtained by confocal microscopy, are shown. A, apical surface; B, basal surface. (B) MDCK cells expressing MT1F were subjected to the collagen-film degradation assay as described in Materials and Methods. Cells grown on the collagen film were treated with or without HGF and with or without GM6001 for 24 h. Degradation of the collagen film was analyzed using wide-field light microscopy. (C) MDCK cells cultured on Transwell inserts were treated with or without HGF for 24 h. Cells were then fixed and stained for ZO-1 (green) and actin (red). (D) The percentage of MT1-MMP that localized at the apical and basal surfaces in A were analyzed as described in Materials and Methods. Twenty confocal images were analyzed for each treatment. ***P<0.0001.

Polarized epithelial cells are in contact with the ECM only at the basal surface. Therefore, in order for epithelial cells to degrade the ECM, it is essential that they localize the ECM-degrading enzyme MT1-MMP to the basal membrane. When cells are cultured in a collagen gel or on a collagen film, MT1-MMP expression is induced, but the cells do not invade or degrade the collagen matrix (Kadono et al., 1998). It has also been reported that MDCK cells that overexpress MT1-MMP show little invasive activity, unless they are treated with HGF (Hotary et al., 2000). These reports suggest that MT1-MMP activity in epithelial cells is predominantly regulated post-translationally. To gain further insight, we first examined the localization of MT1-MMP in different membrane domains of MDCK cells that were cultured on a thin film of fibrillar collagen on a Transwell inserts until they became confluent and had established epithelial polarity. Cells were then treated with or without HGF (50 ng/ml) for 24 h. Because the antibody we used against human MT1-MMP does not recognize endogenous canine MT1-MMP in immunofluorescence assays, we transduced MDCK cells with the gene encoding FLAG-tagged human MT1-MMP (MT1F) by using adenovirus and examined the localization of MT1F. As shown in Fig. 1A, without HGF treatment, MT1F localized exclusively at the apical surface of well-polarized MDCK cells, suggesting that the default secretory pathway of MT1-MMP-containing vesicles is towards the apical surface. This would effectively dissociate MT1-MMP from the ECM and, under these conditions, cells exhibit only minor collagenolytic activity – although they expressed MT1F (Fig. 1B, Cont). However, when these cells were stimulated with HGF, a significant amount of MT1F was detected at the basal surface of the cells (Fig. 1A), resulting in extensive degradation of collagen (Fig. 1B, HGF). The collagenolytic activity of the cells was completely inhibited by addition of the general metalloproteinase inhibitor GM6001. Treatment with HGF did not alter the integrity of tight junctions, as seen by staining for the tight junction protein ZO-1 staining (Fig. 1C), which suggests that the appearance of MT1F at the basal surface is not due to the loss of epithelial phenotype and/or polarity. The amount of MT1F localized at the basal surface was about 20% of the total amount of MT1-MMP on the cell surface in this experiment (Fig. 1D), which is sufficient to exhibit collagenolytic activity.

**Fig. 2.**
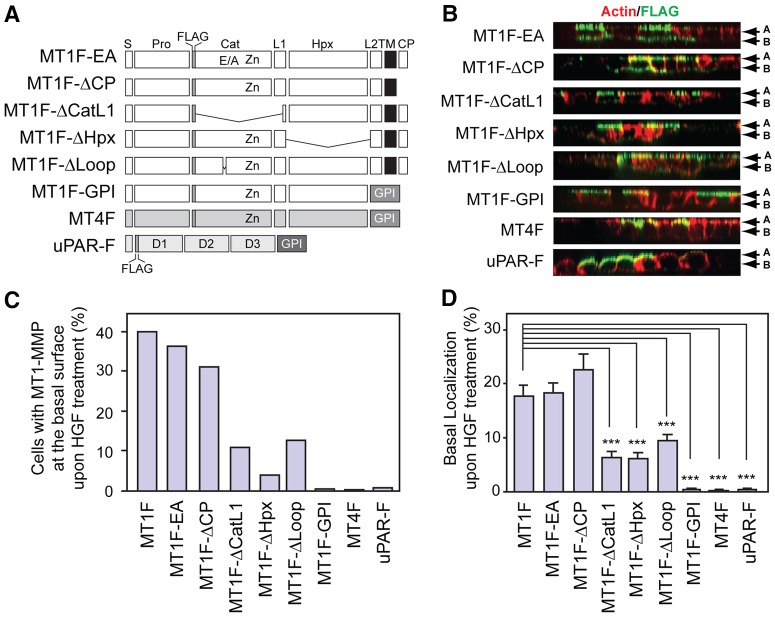
**Basal localization of MT1-MMP mutants and GPI-anchored proteins.** (A) Constructs used in the experiments. All constructs had been tagged with the FLAG epitope in their ectodomain. S, signal peptide; Pro, prodomain; Cat, catalytic domain; L1, linker 1 region; Hpx, hemopexin domain; L2, linker 2 region; TM, transmembrane domain; CP, cytoplasmic domain; FLAG, FLAG epitope; Zn, catalytic zinc ion; GPI, GPI-anchoring signal; D1, D2 and D3, domain 1–3 of uPAR. (B) MDCK cells expressing the MT1-MMP mutants and GPI-anchored proteins shown in A were treated with HGF for 24 h and the cell surface localization of these proteins was analyzed as in Fig. 1. Representative images of XZ cross-sections of cells expressing each protein are shown. A, apical; B, basal surface. (C) The percentage of the cells in which the constructs localized at the basal surface was analyzed for 200 cells positive for the FLAG-tag signal. (D) The proportion of MT1-MMP localized at the basal surface in B was analyzed as in Fig. 1D. ***P<0.0001. At least 20 confocal image files for each condition were incorporated into the analysis.

To examine which domains of MT1-MMP are required for this change in the localization of MT1F upon HGF treatment, various mutants of MT1-MMP were examined, including the inactive Glu240Ala mutant (MT1F-EA), and mutants that lack the cytoplasmic domain (MT1F-ΔCP), the catalytic domain and L1 region (MT1F-ΔCatL1), the hemopexin domain (MT1F-ΔHpx) and the MT-loop (MT1F-ΔLoop) (Fig. 2A). The MT-loop is an insertion within the catalytic domain characteristic for only transmembrane-type MT-MMPs, namely MT1-, MT2-, MT3- and MT5-MMP (Fernandez-Catalan et al., 1998; Sato et al., 1994). In the absence of HGF, all mutant MT1-MMPs localized exclusively to the apical surface of the plasma membrane in the absence of HGF, as did wild-type MT1-MMP (data not shown). Upon treatment with HGF, the catalytic activity of the enzyme or its cytoplasmic domain were not required for localization of MT1-MMP to the basal membrane, because MT1F-EA and MT1F-ΔCP localized to the basal membrane in a manner similar to wild-type MT1F; both regarding the number of cells in which these MT1-MMP mutants localized to the basal side of the membrane (Fig. 2C) and the proportion of the enzyme that localized to the basal side in these cells (Fig. 2D). However, localization of MT1F-ΔCatL1, MT1F-ΔLoop and MT1F-ΔHpx was significantly less pronounced on the basal membrane than that of MT1F. Because the MT-loop is the 8-amino-acid loop in the catalytic domain, the MT-loop region is likely to be responsible for the catalytic-domain-dependent change in localization. These data suggest that the effective switchover of MT1-MMP localization requires both the catalytic and the Hpx domains.

Glycosylphosphatidylinositol (GPI)-anchored proteins are exclusively secreted to the apical surface of polarized epithelial cells (Lisanti and Rodriguez-Boulan, 1990). Therefore, we examined the localization of two GPI-anchored proteins uPAR and MT4-MMP, uPAR and MT4F, respectively, when FLAG tagged, under the same conditions (Fig. 2A). Both localized solely at the apical surface of inert MDCK cells (data not shown) and treatment with HGF did not modify their apical localization (Fig. 2B-D). This result further confirmed that treatment with HGF did not cause a loss of epithelial polarity in this experimental system. Interestingly, when localization of chimeric GPI-anchored MT1-MMP (MT1F–GPI) was examined (Fig. 2A), we found that MT1F–GPI localized exclusively at the apical surface regardless of HGF treatment (Fig. 2B-D), suggesting that GPI-anchoring overrides the ectodomain-dependent switch of MT1-MMP localization.

### Localization of MT1-MMP in the epithelial tubule within 3D collagen gel

**Fig. 3.**
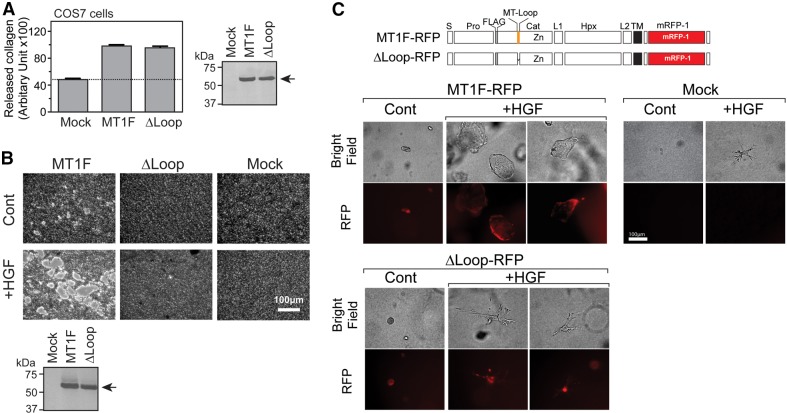
**Relevance of basal localization of MT1-MMP in collagen degradation, and invasion by MDCK cells.** (A) COS-7 cells were transiently transfected with expression plasmids for MT1F, MT1F-ΔLoop (ΔLoop) or empty vector. Cells were then subjected to the 3D-collagen-degradation assay as described in the Materials and Methods (*n* = 4). Expression of MT1F and ΔLoop in these COS-7 cells was confirmed by western blotting using the FLAG M2 antibody (right panel). Note that both MT1F- and ΔLoop-expressing COS-7 cells degraded collagen at similar levels. (B) MDCK cells stably expressing MT1F or MT1F-ΔLoop were established. Cell lysates were subjected to western blot analysis using the FLAG M2 antibody (lower panel). These cells were subjected to the collagen-film degradation assay described in the Materials and Methods. Cells grown on the collagen film were treated with or without HGF for 24 h and degradation of the collagen film was analyzed by wide-field light microscopy (upper panels). Scale bar: 100 µm. (C) The top panel shows a schematic representation of the constructs used in the experiments. MT1F and MT1F-ΔLoop were fused with mRFP-1 at their cytoplasmic domain (MT1F-RFP and ΔLoop-RFP, respectively) as described in the Materials and Methods. MDCK cells stably expressing MT1F-RFP or ΔLoop-RFP or Mock-transfected cells were subjected to 3D collagen culture for 5 days in the presence or absence of HGF. Representative area of images in the brightfield or RFP channel (wide-field fluorescence images) are shown. Scale bar: 100 µm. Cont, control.

The MT1F-ΔLoop mutant is defective in basal localization (Fig. 2); therefore, this mutant might be useful to examine the biological significance of basal localization of MT1-MMP. The mutant has been previously characterized and it was shown that the mutation has no effect on the catalytic function of MT1-MMP (English et al., 2001). We also examined whether deletion of the MT-loop has any effect on the collagenolytic activity of MT1-MMP (Fig. 3A). In these experiments, COS-7 cells were transfected with the expression plasmid encoding MT1F or MT1F-ΔLoop and subjected to the 3D-collagen-degradation assay. Our data indicated that MT1F and MT1F-ΔLoop showed similar levels of expression and that they degraded a comparable amount of collagen, suggesting that the main difference between MT1F and MT1F-ΔLoop is the ability of the two proteins to localize to the basal surface. Therefore, we used the MT1F-ΔLoop mutant to examine the functional relevance of the basal localization of MT1-MMP in epithelial cells. We thus established MDCK cells that stably express MT1F or MT1F-ΔLoop and subjected the cells to the collagen-degradation assay (Fig. 3B). Inert MT1F-expressing MDCK cells showed a small amount of collagen degradation but treatment with HGF induced increased collagen degradation. However, cells expressing MTF1-ΔLoop showed no obvious collagen degradation, regardless of HGF treatment. As MT1F-ΔLoop can degrade collagen to an extent similar to the wild-type enzyme (Fig. 3A), this phenotype is due to the lack of basal localization of this mutant in MDCK cells.

Next we addressed the importance of basal localization in 3D collagen culture, where they undergo tubulogenesis within 3D collagen in an MT1-MMP-dependent manner (Kadono et al., 1998; supplementary material Fig. S1). MDCK cells overexpressing MT1F tagged with mRFP-1 (MT1F-RFP) (Itoh et al., 2011), or mRFP-1-tagged MT1F-ΔLoop (ΔLoop-RFP) were cultured in 3D collagen for 5 days in the presence of HGF. Under these culture conditions, the lumen side of the tubule or cyst is the apical surface, and the collagen-attaching side is the basal surface. Thus, the enzyme would not be able to degrade the surrounding collagen matrix without localizing to the basal side. mRFP-1 was inserted into the cytoplasmic domain of MT1-MMP after cysteine residue 574 (Cys574) with a linker of two glycines and the rest of the cytoplasmic domain of MT1-MMP (Gln575–Val582) was attached to the C-terminus of mRFP-1. We have previously confirmed that this chimeric enzyme retains all of the tested biological activities of MT1-MMP (Itoh et al., 2011). As shown in Fig. 3C, without treatment with HGF, cells expressing MT1F-RFP or ΔLoop-RFP formed slightly larger cysts than cells expressing the mock vector (Mock). Upon HGF treatment, MDCK cells overexpressing MT1F-RFP formed large cysts, whereas mock-treated cells formed tubules (Mock, +HGF) (Fig. 3C, MT1F-RFP, +HGF). This phenotype is in agreement with a previous report (Kadono et al., 1998) and it is due to excess degradation of the collagen matrix by MT1F-RFP. However, MDCK cells overexpressing ΔLoop-RFP showed normal tubule phenotypes. Expression of ΔLoop-RFP in these cells was confirmed to be similar to that of MT1F-RFP, as shown in the channel detecting RFP fluorescence. Thus, the inability of ΔLoop-RFP to convert tubules to cysts is most likely due to lack of basal localization of the enzyme. These data suggest that basal localization of MT1-MMP is an important factor that influences epithelial cell morphogenesis in a 3D collagen matrix.

**Fig. 4.**
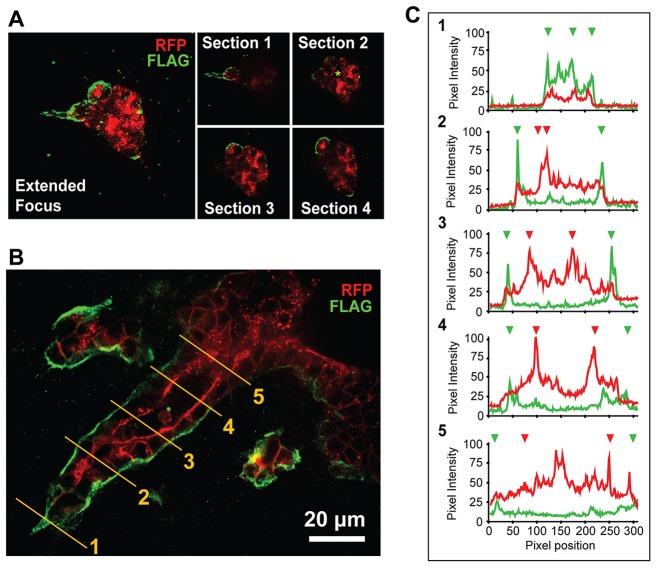
**Localization of MT1-RFP to the basal surface of MDCK cells undergoing branching-morphogenesis.** MDCK cells that stably express MT1F-RFP were cultured in a 3D collagen gel (2 mg/ml) in the presence of HGF (50 ng/ml) for 3 or 6 days. Cells in the gel were fixed and subjected to whole-mount immunofluorescence staining using the FLAG M2 antibody without permeabilization, as described in the Materials and Methods. (A) Cells were fixed after 3 days, stained with the FLAG M2 antibody (green) and analyzed using confocal microscopy. The left panel is an extended focus image combining all confocal sections and the four smaller panels on the right are single sections from different focuses, 15 µm apart from the top (Section 1) towards the bottom (Section 4) to highlight the cells with a FLAG signal (green fluorescence) and the presence of lumen. The red signal is derived from MT1F-RFP. * indicates the lumen in the cyst. (B) Cells were fixed after 6 days. A single-confocal-section image is shown. (C) Fluorescence intensities of both the green and red channels at the position indicated in B are shown. Green and red inverted triangles indicate the position of basal and apical membrane, respectively.

**Fig. 5.**
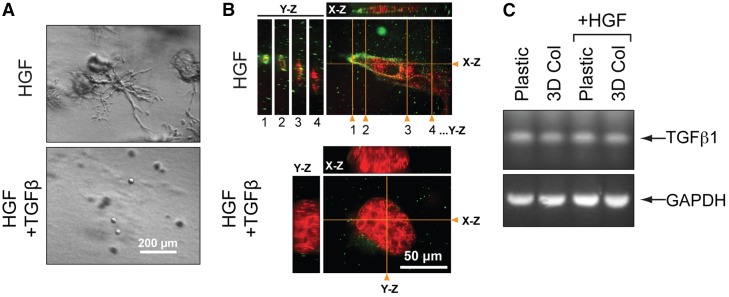
**Effect of TGFβ on the tubulogenesis of MDCK cells.** (A) MT1F-RFP-expressing MDCK cells were cultured in the presence of HGF (50 ng/ml) with or without TGFβ (10 ng/ml) for 7 days. Note that TGFβ treatment completely abolished tubulogenesis of MT1-RFP-exressing MDCK cells. (B) MDCK cells in the collagen gel in A were subjected to whole-mount immunofluorescence staining using the FLAG M2 antibody, as described for Fig. 3. Positions of Y-Z and X-Z cross sections are indicated with orange lines and triangles. (C) Expression of the gene encoding TGFβ1 by using PCR in MDCK cells cultured on plastic or collagen film (3D Col) in the presence or absence of HGF (50 ng/ml) for 3 days. Amplification of GAPDH was taken as a control.

Expression of high levels of MT1F-RFP in MDCK cells destroyed tubules, as shown in Fig. 3C; however, cells that expressed lower levels of MT1F-RFP are still able to form tubules, although the tubules are larger than those of non-transfected MDCK cells. We postulated that exogenously expressed MT1-MMP levels in this population of cells are low enough to be regulated by the cells and that analyzing localization of exogenous MT1-MMP might represent endogenous enzyme localization. Therefore, we next addressed whether there are different levels of MT1-MMP at the basal surface of tubules formed in 3D collagen. We chose an MDCK cell population that expresses MT1F-RFP in moderate levels and confirmed that they formed smooth but larger tubules in 3D collagen upon stimulation with HGF (Figs 4, 5). Cells were grown within 3D collagen gel in the presence of HGF for 3 or 6 days; tubules in the collagen gels were then fixed and stained with antibody against FLAG without prior permeabilization. This way, the antibody stains only MT1F-RFP localized at the basal side because IgG cannot diffuse into the apical lumen side owing to the presence of the tight junctions. At day 3, cells formed a small cyst and extended a protrusion into the collagen matrix (Fig. 4A). MT1F-RFP was expressed in all the cells, as detected by RFP signals, but basally localized MT1F-RFP (green) was found in only a few cells, including those with a protrusion. They had already formed a lumen, as indicated by the sections obtained by confocal microscopy (Fig. 4A, section 2, *). At day 6, the tubule was clearly formed, and again every cell maintained MT1F-RFP expression across the tubule (red signals, Fig. 4B). However, basally localized MT1-MMP (green) showed a polarized distribution, having a higher intensity towards the tip compared with the base of the tube. Green signals at the base were approximatey one-fifth less intense compared with green signals at the tip of the tube structure, despite having a similar overall red signal intensity (Fig. 4B,C). These data suggest that basal localization of MT1-MMP, indeed, occurs during tubulogenesis and that localization seems to be correlated with the extension of tubule structures into a collagen matrix.

### Bi-phasic role of TGFβ in tubulogenesis

As shown above, HGF is a well-known positive morphogen that modifies the shape of MDCK cells in 3D collagen. Another morphogen that is known to modify epithelial tubulogenesis is TGFβ (Nelson et al., 2006; Santos et al., 1993), which has been shown to inhibit tubulogenesis of MDCK cells (Santos et al., 1993). Therefore, we next asked if TGFβ-dependent inhibition of tubulogenesis correlates with basal localization of MT1-MMP. When a population of MDCK cells that expresses MT1F-RFP was cultured in the presence of HGF, we found that large tubules (HGF) and some large cysts (HGF + TGFβ) formed in 3D collagen gel (Fig. 5A). We confirmed that the tips of the tubule structure contained basal MT1-RFP, as shown in Fig. 5B (HGF). Interestingly, when these cells were cultured in the presence of HGF and TGFβ (10 ng/ml), they did not form any tubules or large cysts but remained as small cysts (Fig. 5A). Although MT1F-RFP was still expressed in these cells – because all cells were positive for RFP signals – the enzyme was not detected at the basal side of the membrane, as shown by the lack of green signal (Fig. 5B). This suggests that TGFβ is a negative regulator of MT1-MMP localization to the basal surface and that this is a mechanism whereby TGFβ inhibits tubule formation. MDCK cells have been shown to express TGFβ (Wyatt et al., 2007) and we confirmed that they spontaneously expressed the TGFβ1 isoform, regardless of whether the cells were stimulated with HGF or cultured on the collagen film (Fig. 5C). If endogenous TGFβ could inhibit basal localization of MT1-MMP, asymmetrical TGFβ signaling can dictate which cells invade into collagen for tubulogenesis, as proposed by Nelson et al. (Nelson et al., 2006).

**Fig. 6.**
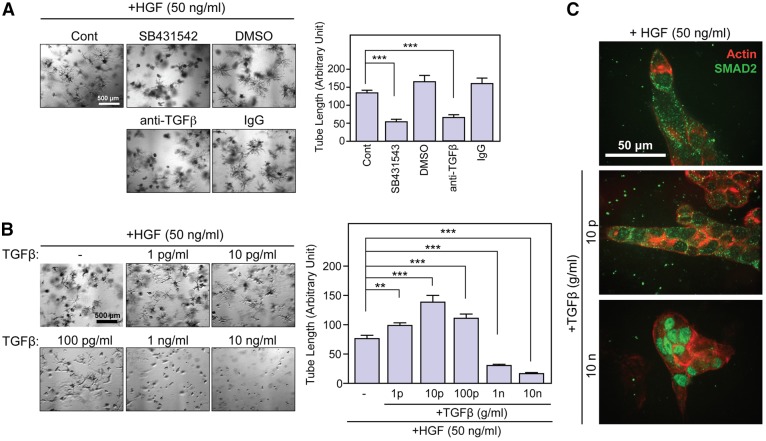
**Bi-phasic effect of TGFβ on tubulogenesis of MDCK cells.** (A) MDCK cells were cultured in 3D collagen in the presence of HGF with or without the inhibitor of TGFβ receptor tyrosine kinase SB431542 (10 µM) or pan-neutralizing antibody against TGFβ (anti-TGFβ, 1 µg/ml) for 7 days. The length of tubes was measured (*n* = 30) and data were plotted in the right panel. Mean±s.e.m. are shown. ***P<0.0001. (B) MDCK cells were cultured in 3D collagen in the presence of HGF with the indicated concentrations of TGFβ. The length of tubes was measured (*n* = 30) and plotted in the right panel. Mean±s.e.m. are indicated. **P = 0.034, ***P<0.0005. (C) MDCK cells were cultured in 3D collagen gel in the presence of HGF with or without TGFβ (10 pg/ml or 10 ng/ml) for 7 days. Cells were fixed, permeabilized and subjected to whole-mount immunofluorescence staining for SMAD2 (green). Cells were also counter-stained for actin (red).

Following the results above, we attempted to understand the role of endogenous TGFβ by blocking TGFβ signaling. We used two different approaches to inhibit endogenous TGFβ: one that used the TGFβ receptor tyrosine kinase inhibitor SB431542, and one that used a pan-TGFβ neutralizing antibody. If endogenous TGFβ is acting as a negative regulator, these inhibitor treatments should result in exaggerated tubule structures or formation of large cysts. However, the results indicated that blocking of TGFβ signaling negatively affected tube formation (Fig. 6A), causing overall decreased length of tubules. This suggests that endogenous TGFβ is not acting as an inhibitor but, rather, that it is necessary for efficient tubule formation in MDCK cells. Subsequently, we treated MDCK cells with different concentrations of exogenous TGFβ ranging from 1 pg/ml to 10 ng/ml. The data indicated that TGFβ had a bi-phasic action. At concentrations of 10–100 pg/ml it enhanced tubulogenesis; however, at 1–10 ng/ml, it inhibited this process (Fig. 6B). To examine if a SMAD2-dependent canonical signaling pathway is involved in the bi-phasic action of TGFβ, localization of SMAD2 was analyzed in tubules grown in 3D collagen in the presence or absence of exogenous TGFβ (Fig. 6C). The data indicated that in the absence of exogenous TGFβ, or upon addition of 10 pg/ml of TGFβ, SMAD2 was mostly localized in the cytoplasm, whereas treating cells with 10 ng/ml of TGFβ resulted in the exclusive localization of SMAD2 to the nucleus. This indicates that the inhibitory effects of TGFβ on tubulogenesis are mediated through the canonical pathways of TGFβ signaling; however, the tube-formation-enhancing effect at lower doses of TGFβ is likely to be mediated through the non-canonical pathways.

**Fig. 7.**
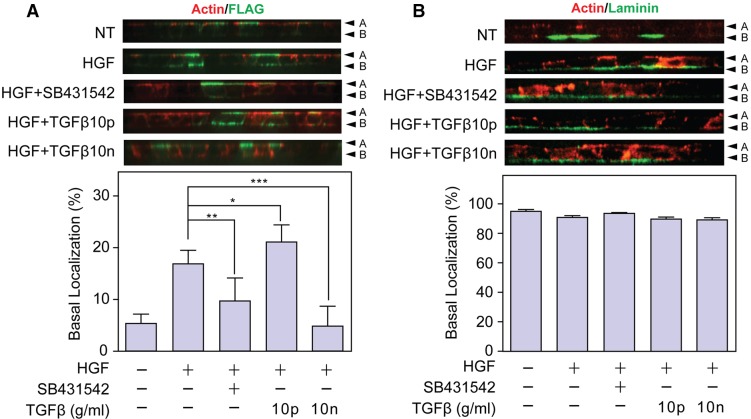
**TGFβ signaling affects basal localization of MT1-MMP.** (A) MDCK cells expressing MT1F were cultured on collagen-coated Transwell inserts and treated with or without HGF (50 ng/ml) in the presence or absence of SB431542, and TGFβ (10 pg/ml or 10 ng/ml) for 24 h. Cells were stained for MT1F (green) and actin (red). The proportion of basally localized MT1-MMP was analyzed, as in Fig. 1D (*n* = 30) (lower panel). Mean±s.e.m. are shown. *P = 0.02, **P = 0.0015, ***P<0.0001. (B) Nontransfected MDCK cells were cultured on collagen-coated Transwell inserts and treated with or without HGF (50 ng/ml) in the presence or absence of SB431542, and TGFβ (10 pg/ml or 10 ng/ml) for 24 h. Cells were stained for laminin (green) and actin (red). The proportion of basally localized laminin was analyzed as in Fig. 1D (*n* = 5) (lower panel). Note that there is no significant change in localization of laminin at the basal surface in different treatments. A, apical surface; B, basal surface; NT, nontransfected.

To further examine whether these tubule phenotypes correlate with basal membrane localization of MT1F, we examined the effect of exogenous TGFβ in the Transwell culture system (Fig. 7A). At 10 ng/ml TGFβ inhibited HGF-induced basal localization of MT1F, whereas at 10 pg/ml TGFβ basal localization was enhanced. Treatment with SB431542 decreased basal localization of MT1-MMP significantly (Fig. 7A). To examine whether TGFβ specifically inhibits the basal localization of MT1-MMP, or whether TGFβ has a general effect on basal-localizing proteins, we investigated localization of laminin (laminin α1 chain), a component of the basal lamina. As shown in Fig. 7B, more than 90% of laminin was detected at the basal surface, regardless of treatment with HGF, SB431542 or TGFβ, suggesting that the inhibitory effect of TGFβ on the localization of MT1-MMP to the basal surface is not part of a general effect on basal secreting proteins but is, instead, specific to MT1-MMP. Taken together, these data suggest that TGFβ modifies epithelial morphogenesis by affecting MT1-MMP localization between the apical and the basal surfaces.

To investigate whether endogenous MT1-MMP is regulated in a similar manner, we also examined mouse mammary gland epithelial cells (NMuMG) that, when cultured in 3D collagen gel, formed tubule structures even without HGF stimulation (supplementary material Fig. S2A). However, treatment with HGF further enhanced tubulogenesis. The tubulogenesis was effectively inhibited by either DX-2400, the highlyselective inhibitory antibody against MT1-MMP (Devy et al., 2009), or GM6001, suggesting that tubulogenesis of NMuMG cells is also MT1-MMP dependent. As shown in supplementary material Fig. S2B, tubulogenesis of NMuMG cells was effectively inhibited by high doses of TGFβ, regardless of stimulation with HGF. In contrast to MDCK cells, however, SB431542 did not inhibit tubulogenesis, suggesting that endogenous TGFβ signaling is not involved in tubulogenesis of NMuMG cells. When these cells were subjected to collagen-film degradation assay, it became evident that the ability of cells to form tubules correlated well with the degradation of the collagen film (supplementary material Fig. S2C). Overall levels of MT1-MMP, in the NMuMG cells cultured on a film of collagen was not affected by treatment with HGF or TGFβ (supplementary material Fig. S2D), suggesting that the inhibition of collagen-film degradation by TGFβ is most likely due to inhibition of the basal localization of MT1-MMP. These data strongly support the idea that apical-basal localization of MT1-MMP is a crucial mechanism that influences epithelial morphogenesis in a collagen-rich environment. Attempts to detect localization of endogenous MT1-MMP in NMuMG cells were hampered by its low protein levels and the lack of sensitivity of the antibody used in the immunofluorescence assays.

We further examined human mammary epithelial (MCF10A) cells to confirm a role of endogenous TGFβ. MCF10A0020 cells formed tubule structures in the collagen gel upon treatment with HGF that, again, was MT1-MMP dependent because it could be inhibited by DX-2400 and GM6001 (supplementary material Fig. S3A). Tubulogenesis of MCF10A cells was also inhibited by addition of TGFβ in a dose-dependent manner; and SB431542 also inhibited tubulogenesis in these cells, as observed for MDCK cells (supplementary material Fig. S3B). Again, it was impossible to detect endogenous MT1-MMP in immunofluorescence assays.

## DISCUSSION

It has been shown that MT1-MMP is localized to the leading edge of different migratory and/or invasive cells, including to the lamellipodia (Mori et al., 2002), podosomes (Wiesner et al., 2010), collagen attachment sites (Bravo-Cordero et al., 2007), focal adhesion sites (Wang and McNiven, 2012) and invadopodia of invasive cancer cells (Nakahara et al., 1997; Poincloux et al., 2009). However, it has not been clear how MT1-MMP localization is regulated in multicellular structures. Here, we report that MT1-MMP localization between the apical and the basal surfaces of an epithelial layer is regulated to facilitate extension of a multicellular-tube structure in 3D collagen. The default pathway of MT1-MMP secretion in inert polarized epithelial cells is towards the apical membrane surface, and localization is partially switched to the basal side of the membrane upon treatment with HGF. Basal localization of MT1-MMP is essential for epithelial cells to degrade the surrounding collagen matrix during the morphogenic program. Taken together, these data suggest a novel paradigm for the regulation of MT1-MMP that provides an additional level of spatiotemporal control in epithelial invasion into the surrounding extracellular matrix.

Polarized protein secretion in epithelial cells is regulated by vesicle transport, and the switch of MT1-MMP localization from the apical to the basal surface is likely to be attributed to the use of alternative vesicle-trafficking pathways. There are several potential scenarios to make this switch of MT1-MMP localization possible. First, exocytosis could be altered. In this model, HGF stimulation might result in the re-sorting of MT1-MMP into vesicles that are targeted to the basal surface, or HGF could modify the interaction of adaptor and/or motor proteins at the outer leaflet of MT1-MMP-containing vesicles. Second, retention time at the basal surface might have been altered. It is possible that some MT1-MMP is constitutively secreted to the basal surface of inert epithelial cells but that consistent and rapid endocytosis of MT1-MMP leads to low basal levels of MT1-MMP. Treatment with HGF might trigger inhibition of this rapid endocytosis process by inducing expression of a protein that interacts with MT1-MMP at the basal surface; thereby, leading to accumulation of MT1-MMP. Third, enhanced transcytosis of MT1-MMP might be a mechanism. It has been previously reported that 80% of endocytosed MT1-MMP molecules in MDCK cells are recycled back to the cell surface (Wang et al., 2004). It is thus possible that MT1-MMP endocytosed from the apical surface is sorted again in the endosome into vesicles that are targeted to the basal surface. Regardless of the specific mechanism, sorting of MT1-MMP into vesicles that target to the basal surface is probably key to regulating the localization of MT1-MMP to the basal surface. We have previously reported that the cytoplasmic domain of MT1-MMP interacts with adaptor protein 2 (also known as AP2A2) to initiate endocytosis through a clathrin-dependent mechanism (Uekita et al., 2001). However, as the cytoplasmic domain of MT1-MMP is dispensable for the basal localization, sorting of MT1-MMP into vesicles is not due to a direct interaction of MT1-MMP with cytoplasmic adaptor proteins. We found the basal localization of MT1-MMP to be dependent on the ectodomain of MT1-MMP, including the 8-amino-acid MT-loop in the catalytic domain, and the Hpx domain, suggesting that the interaction with other molecule(s) through the ectodomain of MT1-MMP is important for the sorting of MT1-MMP into basal-targeting vesicles. It has been reported that the Rab8 GTPase-dependent exocytic pathway regulates the pro-invasive activity of MT1-MMP in human breast carcinoma cells MDA-MB231 (Bravo-Cordero et al., 2007). It was also shown that Rab8a, Rab14 and Rab22a are involved in secretion of MT1-MMP in macrophages (Wiesner et al., 2013) and that Rab8 is involved in exocytic pathways of basolateral-membrane proteins in MDCK cells (Henry and Sheff, 2008; Huber et al., 1993). Thus, it is possible that ectodomain interaction of MT1-MMP with another molecule(s) plays a role in sorting MT1-MMP into vesicles that are secreted to the basal surface in a Rab8-dependent manner.

Our data showed that efficient transport of MT1-MMP-containing vesicles to the basal surface requires both HGF and endogenous TGFβ in MDCK cells. Stimulation with HGF in the presence of SB431542 could not efficiently direct MT1-MMP to the basal surface and could not stimulate tubule formation in an efficient manner (Fig. 6). Interestingly, the effect of exogenous TGFβ was bi-phasic: it enhanced the basal localization of MT1-MMP at lower concentrations (10 pg/ml) but inhibited at higher concentrations (10 ng/ml). This correlates well with the ability of TGFβ to stimulate tubulogenesis as TGFβ enhanced tubulogenesis at 1–100 pg/ml and inhibited it at 1–10 ng/ml. Addition of TGFβ alone at any concentration induced neither the localization of MT1-MMP to the basal surface nor tubulogenesis (data not shown). Thus, both HGF and TGFβ signaling are essential for MT1-MMP localization and efficient tubulogenesis. The bi-phasic effect of TGFβ has been previously reported. In breast cancer cells, 1–50 pg/ml of TGFβ enhanced growth whereas 1 ng/ml suppressed growth (Welch et al., 1990). In bovine microvascular endothelial cells, *in vitro* angiogenesis was enhanced by TGFβ at 100 pg/ml–1 ng/ml and inhibited at 5–10 ng/ml (Pepper et al., 1993). Interestingly, TGFβ commonly enhances cellular invasion at lower doses and inhibits it at higher doses. We found that at a higher concentration TGFβ signals through the canonical pathway, whereas at lower doses signaling is mediated through SMAD-2-independent non-canonical pathways.

TGFβ is commonly regarded as a negative morphogen for epithelial morphogenesis (Nelson et al., 2006; Santos et al., 1993). It has been shown that mammary epithelial cells constitutively produce TGFβ, and that areas of epithelial structures with higher local levels of endogenous TGFβ suppressed tubulogenesis, whereas areas with lower levels extended tubule structures into the collagen gel (Nelson et al., 2006). However, the levels of active endogenous TGFβ in the MDCK cell culture system were not high enough to exhibit an inhibitory effect but were sufficient to enhance tubulogenesis. We also observed enhanced tubulogenesis when MDCK cells were seeded more densely in the 3D collagen gel (1×10^5^ cells/ml compared with 1×10^4^ cells/ml), which is likely to cause localized increased levels of active endogenous TGFβ within the culture (data not shown). We speculate that local availability of active TGFβ across the population of cells that are forming a structure determines which population of cells extend the structure into the collagen matrix, and that this is, at least in part, attributed to the localization of MT1-MMP to the basal surface. TGFβ signaling is uniquely regulated post-translationally by activation of latent TGFβ, which forms a complex with latent TGFβ binding protein 1 (LTBP1), through the action of proteinases, integrin or thrombospondin (Keski-Oja et al., 2004). It is not clear which of these mechanisms plays a role during tubulogenesis but it is unlikely that metalloproteinases are involved because we observed TGFβ-dependent basal localization of MT1-MMP in the presence of GM6001 (Fig. 6). Further investigation of the local activation of TGFβ across the epithelial cell layers are important to understand the mechanism of epithelial morphogenesis.

Interestingly, the positive role of endogenous TGFβ in tubulogenesis seems to be cell-line-specific. Our data indicate that NMuMG cells do not require endogenous TGFβ signaling for tubulogenesis as addition of SB431542 had no effect on tubulogenesis (supplementary material Fig. S2). However, both in MDCK and MCF10A cells, blocking the signaling of endogenous TGFβ using SB431542 inhibited tubulogenesis (Fig. 6 and supplementary material Fig. S3). Nevertheless, our data indicate that the level of endogenous TGFβ in at least three epithelial cell lines is not high enough to act as a negative morphogen.

Our findings have established a novel and fundamental mechanism of tubulogenesis in which tubule development is dependent on the localization of the membrane-bound collagenase MT1-MMP to the basal surface of epithelial cells. This mechanism could play a role during the development of epithelial organs, such as submandibular glands, because it has been shown that MT1-MMP is important in forming these structures (Oblander et al., 2005). It is also possible that this mechanism is necessary during angiogenesis and during invasion of well-differentiated epithelial tumor cells where the role of MT1-MMP is well documented. In a well-differentiated colon cancer, MT1-MMP was found to localize at both the apical and the basal surfaces (Murai et al., 2004), suggesting that these cells were stimulated to switch the localization of MT1-MMP over to the basal surface. Thus, understanding the regulatory mechanism of this change in localization of MT1-MMP might shed light on the complex process of epithelial morphogenesis and invasion.

## MATERIALS AND METHODS

### cDNA cloning

FLAG (DYKDDDDK)-tagged MT1-MMP (MT1F), FLAG-tagged human MT4-MMP (MT4F) and uPAR were constructed as described previously (Itoh et al., 1999), and subcloned into pSG5 (Stratagene) and/or pCEP4 (Invitrogen). A FLAG tag was inserted at the end of the propeptide (between Arg111 and Tyr112), so that the activated enzyme has the FLAG tag at its N-terminus. The enzyme can be recognized by an antibody against FLAG (Itoh et al., 1999). MT1F-mRFP-1 is a chimera mutant of MT1F with mRFP-1 (Campbell et al., 2002). The cDNA of mRFP-1 was kindly donated by Roger Y. Tsien (University of California, San Diego, CA). mRFP-1 was inserted into to the cytoplasmic domain of MT1F after Cys574 with a linker of two glycines. The remaining cytoplasmic domain of MT1-MMP (Gln575-Val582) was attached to the C-terminus of mRFP-1 (Itoh et al., 2011). MT1F-EA is a catalytically inactive mutant of MT1F in which Glu240 was mutated to Ala. MT1F-ΔCatL1 is a FLAG-tagged mutant of MT1-MMP in which the region of Tyr112 to Pro312 was deleted (Itoh et al., 2001). MT1F-ΔLoop is a mutant MT1F in which residues Pro163 to Gly170 were deleted (English et al., 2001). MT1F-ΔHpx is a FLAG-tagged mutant of MT1-MMP in which the residues Cys319 to Cys508 were deleted (Itoh et al., 2008). MT1F–GPI is a chimera mutant of MT1F with MT4-MMP in which the corresponding region of Pro509 to Val582 of MT1F was replaced with Glu524 to Leu603 of human MT4-MMP, causing the enzyme to be GPI-anchored (Itoh et al., 1999). MT1F-ΔCP is a cytoplasmic domain (Arg563-Val582) deletion mutant (Uekita et al., 2001). ΔLoop-RFP is a variant of MT1F-RFP lacking Pro163-Gly170. These mutants were generated by the PCR extension method (Ho et al., 1989). All the PCR-generated fragments were confirmed by DNA sequencing and subcloned into pSG5 and/or pCEP4 vector. For MT1F, MT1F-EA, MT1F-ΔCatL1 and MT1F-ΔHpx, adenoviral vectors were also constructed using AdEasyTM system (Q-BIOgene) according to the manufacturer's instructions.

### 2D and 3D culture of epithelial cells

We utilized three epithelial cell lines, including MDCK epithelial cells, NMuMG epithelial cells and MCF10A human mammary epithelial cells. For 2D culture of MDCK cells, the upper surface of Transwell inserts with 8 µm pores (24- or 12-well, BD Biosciences) were coated with a thin layer of neutralized acid-extracted type I collagen (2 mg/ml, Nitta gelatin, Japan) and incubated at 37°C for 30 min to set. Cells were seeded onto the gel and cultured for 48 h until they became confluent and polarized. In some experiments, cells were then infected with adenoviruses to express various genes at a multiplicity of infection (MOI) of 2.5. After 24 h, cells were stimulated with human HGF (50 ng/ml, Peprotech) in the presence of GM6001 (10 µM, Elastin Products Company). Twenty-four hours later, cells were fixed and subjected to immunofluorescence staining. For staining of FLAG at the cell surface, live cells were incubated with the FLAG M2 antibody (2 µg/ml) for 1 h in chilled medium on ice, followed by fixation with 3.3% paraformaldehyde in TBS.

For 3D culture of all epithelial cell lines, cells were suspended in neutralized acid-extracted collagen (2 mg/ml) and incubated for 1 h to allow the collagen to set. Cells were then cultured in the presence or absence of HGF (50 ng/ml) and/or various concentrations of human recombinant TGFβ (R&D Systems) in Dulbecco's modified Eagle's medium containing 10% fetal bovine serum for up to 7 days. In some experiments GM6001, the TGFβ receptor kinase inhibitor SB431542 (10 µM, Sigma-Aldrich), dimethyl sulfoxide solvent control, pan-neutralizing antibody against TGFβ (1 µg/ml, clone 1D11, R&D Systems), DX-2400 (500 nM) or control IgG were also added. DX-2400 is a highly selective MT1-MMP inhibitory antibody with a *K*_d_ of 0.6 nM and was a kind gift from Dyax (Burlington, MA) (Devy et al., 2009).

### Indirect immunofluorescence staining

MDCK cells that had been cultured on collagen-coated Transwell inserts were incubated with the FLAG M2 antibody on ice for 30 min followed by fixation with 3% paraformaldehyde in PBS. Cells were then blocked with 5% goat serum, 3% bovine serum albumin (BSA) in PBS and incubated with Alexa-488-conjugated anti-mouse IgG (Invitrogen) followed by Alexa-568-labeled phalloidin (Invitrogen). For staining of ZO-1, a monoclonal antibody against ZO-1 (Invitrogen) was used. For staining of laminin, a rabbit polyclonal antibody against the laminin α1 chain (Sigma-Aldrich) was used. The membrane was cut out of the plastic well and mounted on a glass slide. Images were captured using the UltraView Confocal Microscope (PerkinElmer) operated by Volocity acquisition software (PerkinElmer).

For staining cells in 3D collagen gels, MDCK cells stably expressing MT1F-RFP were cultured in 100 µl of 3D collagen gel (2 mg/ml) in the presence of HGF (50 ng/ml) to form tubule structures in an 8-well chamber slide for 3 or 6 days. The whole gel in the 8-well chamber was fixed with 3% paraformaldehyde in TBS for 30 min at room temperature and blocked with 5% goat serum and 3% BSA in TBS for 3 h, followed by incubation with a FLAG M1 antibody (5 µg/ml) in the blocking buffer supplemented with 1 mM CaCl_2_ for 18 h at 4°C. After washing the gels three times with TBS, with rocking, for 2 h per wash, Alexa488-conjugated goat anti-mouse IgG (Invitrogen) was incubated in the blocking solution for an additional 18 h. Gels were extensively washed three times in TBS for 3 h per wash, with rocking, and washed once for 14 h. Images were captured using an Ultraview confocal microscope and analyzed by Volocity (Improvision, PerkinElmer) and ImageJ software.

Localization of SMAD2 in MDCK tubules in 3D-collagen was performed under the same conditions but using different antibodies. Rabbit polyclonal antibody against SMAD2 (Abcam, Cambridge, UK) and Alexa-488-conjugated goat anti-rabbit IgG (Invitrogen) were used to detect SMAD2. Cells were also stained for actin with Alexa-568–phalloidin (Invitrogen). Images were captured by using the Ultraview confocal microscope.

### Image acquisition

Images were obtained at room temperature by using a Perkin-Elmer Ultraview LTE Confocal microscope with a Nikon TE-2000 microscope. For fluorescence imaging, a Nikon Plan Apo ×60 oil immersion lens with the numerical aperture (NA) 1.4 was used. For brightfield imaging, a Nikon Plan Fluor ×10 dry lens (NA = 0.3) or Nikon Plan Fluor ×20 dry lens (NA = 0.45) was used. Proteins were labeled using the fluorochromes Alexa 488, Alexa 568 or mRFP-1, as described above. The images were acquired using an Hamamatsu ORCA ER or EMCCD C9100-02 camera and the Volocity Acquisition module (Improvision, PerkinElmer) software.

### Image data analyses

To calculate the proportion of MT1F that localized to the basal surface, fluorescence intensities (FI) of apical and basal surfaces in confocal sections were analyzed by Volocity measurement module software, and the proportion of basal localization (%) was calculated as:
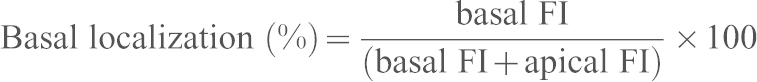
We incorporated the data from at least 20 confocal scanned image files for each construct and/or condition.

To measure the tube length, brightfield images of MDCK cells in collagen gel were captured by using ×4 objective lens on a Nikon TE-2000E microscope equipped with an ORCA-ER CCD camera (Hammamatsu Photonics, Japan) operated by Volocity Acquisition module software. Images were analyzed by ImageJ software to measure the longest tube distance within the structure from one end to the other. Thirty structures per treatment were measured and the results were incorporated into the data.

For both of the above, data were processed using GraphPad Prism to calculate the mean, the standard error of the mean (±s.e.m.), and a two-tailed Student's *t*-test. The experiments were repeated three times, and reproducibility was confirmed.

### Collagen-film degradation assay

Collagen-film degradation assay was performed as described previously (Itoh et al., 2006), with slight modifications. MDCK cells (1×10^6^ cells/well) were cultured on fibrillar collagen in a 6-well plate. Cells were then infected with MT1F adenovirus at MOI of 2.5 and cultured for 48 h in the presence of GM6001 at 10 µM. Medium was changed to serum-free medium without GM6001, with or without HGF. After 24 h of culture, cells were then removed by trypsinization and the remaining collagen film was stained with Coomassie Blue.

To detect endogenous MT1-MMP activity, NMuMG cells (0.75×10^6^) were cultured on fibrillar collagen in a 12-well plate for 24 h in the presence of GM6001 at 1 µM. Cells were then treated with medium containing HGF, TGFβ, SB431542 and/or GM6001, cultured for 3 days, removed by trypsinization and the remaining collagen film stained with Coomassie Blue.

### 3D-collagen-degradation assay

COS-7 cells transiently transfected with MT1F, MT1F-ΔLoop or Mock vector were incorporated into a 3D type-I collagen gel (2 mg/ml, PureCol) containing 50 µg/ml Type-I DQ^TM^ collagen (Life Technologies) at a final cell density of 2.5×10^5^ cells/ml, 200 µl aliquots of the cells were then transferred to Eppendorf tubes. Each gel in the tube was overlayed with 200 µl RPMI-1640 medium without Phenol Red, supplemented with 25 mM HEPES (pH 7) and cultured at 37°C. After 12 h, collagen gels with cells were spun down and the fluorescence intensity of the supernatant was measured (excitation at 488 nm and emission at 520 nm). The average fluorescence and s.e.m. was calculated and plotted.

## Supplementary Material

Supplementary Material
