# Supplementary material for: Basal localization of MT1-MMP is essential for epithelial cell morphogenesis in 3D collagen matrix
Source: J Cell Sci. 2014 Mar 15;127(6):1203–13. doi: 10.1242/jcs.135236 (PMC4117704; doi:10.1242/jcs.135236)
Supplement: Supplementary Material [file supp_127_6_1203__index.html]

Basal localization of MT1-MMP is essential for epithelial cell morphogenesis in 3D collagen matrix — Supplementary Material 

# Basal localization of MT1-MMP is essential for epithelial cell morphogenesis in 3D collagen matrix

## JCS135236 Supplementary Material

**Files in this Data Supplement:**

- **Supplementary Material**
