# Supplementary material for: Basal localization of MT1-MMP is essential for epithelial cell morphogenesis in 3D collagen matrix
Source: J Cell Sci. 2014 Mar 15;127(6):1203–13. doi: 10.1242/jcs.135236 (PMC4117704; doi:10.1242/jcs.135236)
Supplement: Supplementary Material [file supp_127.6.1203_JCS135236.pdf]

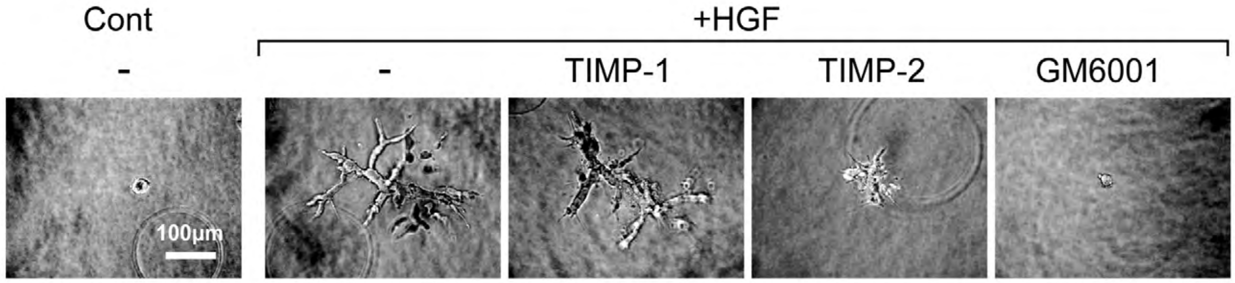

Fig S1. Effect of TIMPs in tubulogenesis of MDCK cells. MDCK cells were subjected to 3D collagen culture in the presence or absence of HGF as described in the Methods section. To investigate involvement of MT1-MMP in this process, they were cultured in the presence or absence of tissue inhibitor of metalloproteinases (TIMP)-1 (500 nM), TIMP-2 (500 nM) and GM6001 (10 µM). Note that TIMP-2 and GM6001 inhibited tubulogenesis, but TIMP-1 did not.

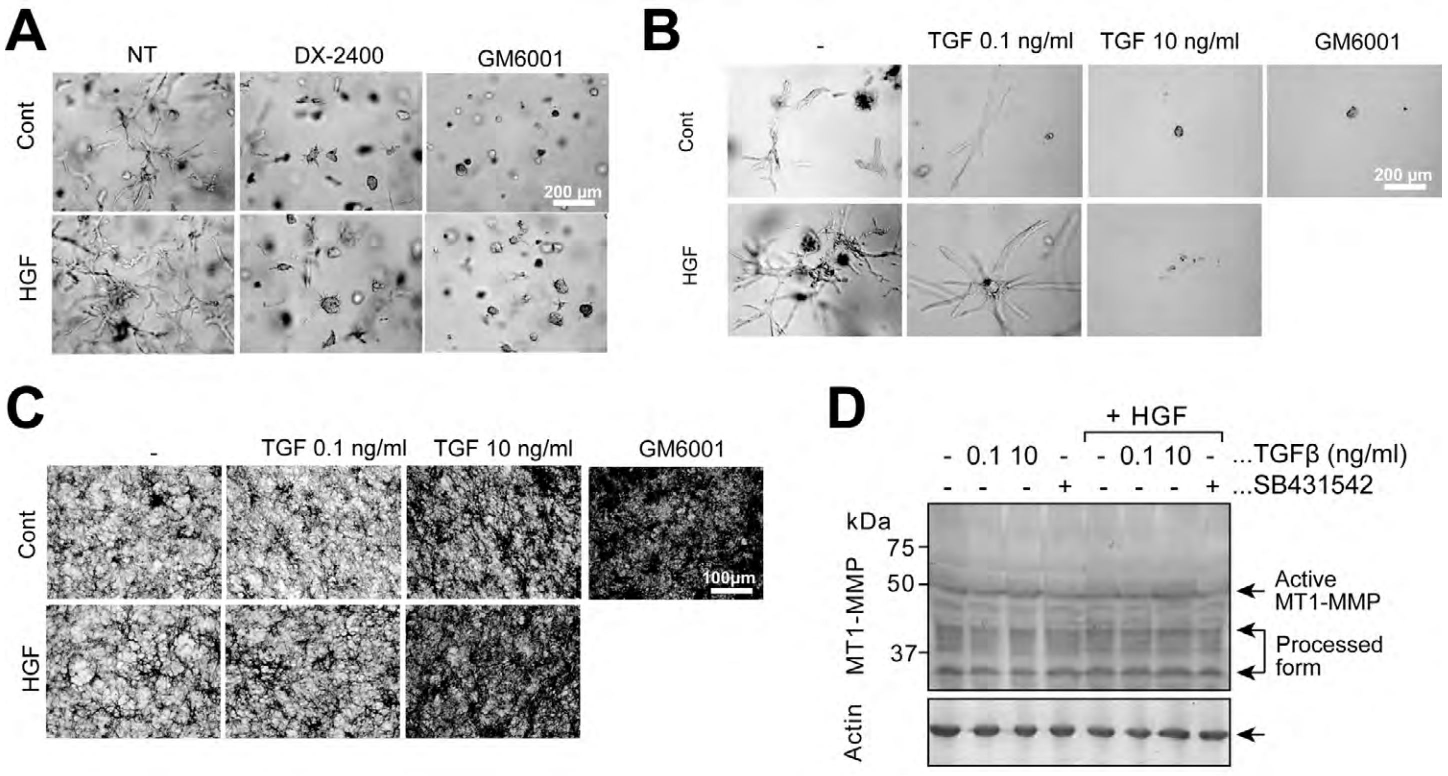

Fig S2. Tubulogenesis and collagen degrading activity is regulated by TGFβ in NMuMG cells. A. Mouse mammary gland epithelial cells NMuMG were subjected to 3D collagen culture in the absence or presence of HGF, DX-2400 (500 nM), and GM6001 (10 µM) for 4 days. Note that DX-2400 and GM6001 effectively inhibited tubulogenesis. **B.** Effect of TGFβ in tubulogenesis of NMuMG cells. Note that TGF β at 10 ng/ml, but not SB431542, inhibited tubulogenesis of NMuMG cells. **C.** NMuMG cells were subjected to collagen film degradation assay as described in the Methods section. Cells were cultured on collagen film for 4 days. Note that TGFβ at 10 nM and GM6001 inhibited collagen film degradation by NMuMG. **D.** Cells on the collagen film as in C were lysed and subjected to western Blot analysis. Note that none of the treatments affected the overall level or pattern of MT1-MMP in NMuMG cells.

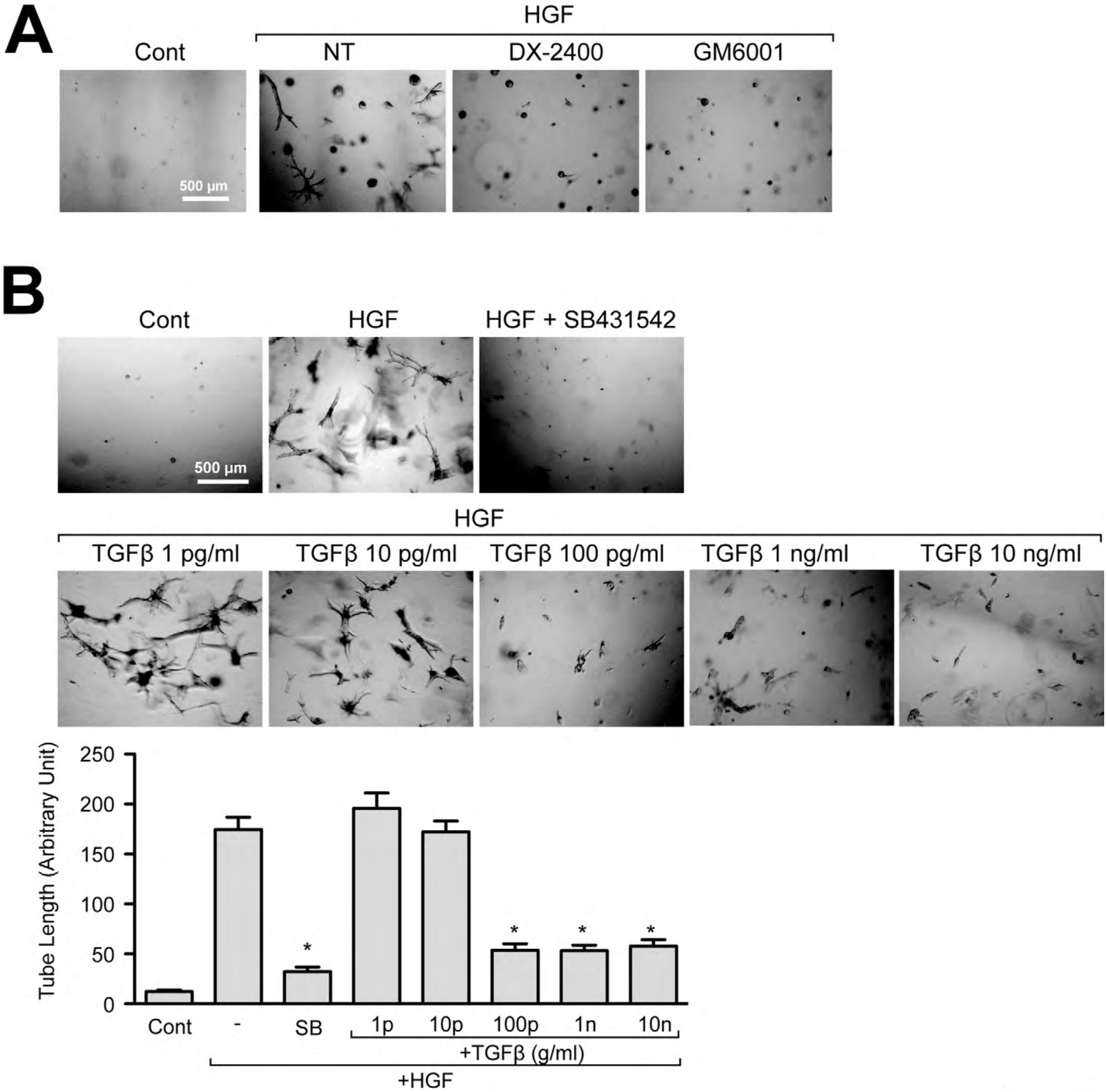

Fig S3. Effect of TGFβ on tubulogenesis by MCF10A cells. A. Human mammary gland epithelial cells MCF10A were subjected to 3D collagen culture in the absence or presence of HGF, DX-2400 (500 nM), and GM6001 (10 μM) for 4 days. Note that DX-2400 and GM6001 effectively inhibited tubulogenesis. **B.** Effect of TGFβ in tubulogenesis of MCF10A cells. Lower panel shows quantitation of length of tubes. Note that TGFβ above 100 pg/ml and SB431542 inhibited tubulogenesis induced by HGF.
